# Impact of admission screening on *Clostridioides difficile* infection rates in a hematology-oncology and hematopoietic cell transplant unit

**DOI:** 10.1017/ice.2026.10442

**Published:** 2026-07

**Authors:** Christine W. Lucky, Lahari Thotapalli, Laura K. Rusie, Yoona Rhee, Michael E. Schoeny, Nicole A. Kraut, Alexandra Seguin, Brian D. Stein, Raul I. Rodriguez, Mary K. Hayden, Michael Y. Lin

**Affiliations:** Rush University Medical Center, Chicago, IL, USA

## Abstract

We evaluated *Clostridioides difficile* admission screening in a cancer unit. Admission screening was associated with a non-significant decrease in hospital-onset *C. difficile* infection (CDI) incidence and a significant decrease in community-onset CDI incidence, which may reflect, in part, the artifactual impact of screening on subsequent provider testing behavior.

## Introduction

*Clostridioides difficile* infection (CDI) disproportionately harms hematology-oncology and hematopoietic cell transplant (HO/HCT) patients due to their immunosuppressed status, prolonged healthcare exposure, and receipt of antimicrobials.^1^ Admission screening for asymptomatic *C. difficile* colonization is a potential strategy to reduce rates of hospital-onset *C. difficile* infection (HO-CDI) because colonized patients, even if asymptomatic, can transmit *C. difficile* to others.^2^ To reduce nosocomial transmission of *C. difficile* in our HO/HCT unit, we implemented *C. difficile* active surveillance for HO/HCT patients at time of admission, placing patients testing positive in isolation precautions. This study assesses the impact of *C. difficile* admission screening on unit-level HO-CDI rates and aims to identify possible unintended impacts of this strategy.

## Methods

Rush University Medical Center (Chicago, IL) is a 678-bed hospital with a 32-bed HO/HCT unit. During the baseline period (May 1, 2020 to May 31, 2022), symptomatic patients with diarrhea underwent *C. difficile* testing of unformed stool by polymerase chain reaction (PCR) [Xpert® *C. difficile/Epi*; Cepheid, Sunnyvale, CA]; asymptomatic patients were not tested. During the intervention period (July 1, 2022 to July 31, 2024), *C. difficile* testing criteria for symptomatic patients remained unchanged,^3^ but asymptomatic patients underwent *C. difficile* colonization screening by PCR [Xpert® *C. difficile*] via perirectal swab at time of admission (testing decision flowchart, Supplemental Figure 1; test results with embedded provider education, Supplemental Figure 2). Patients transferred to the HO/HCT unit from other hospital units were not screened. Patients testing positive for *C. difficile*, whether asymptomatic or symptomatic, were placed in Contact Precautions with hand washing required at time of room exit. Prophylaxis (eg, oral vancomycin) to prevent CDI was not routinely recommended for asymptomatic patients. No other unit-level interventions for CDI prevention were introduced during the study period.

The primary outcome was unit-level HO-CDI incidence (CDI events per 10,000 patient-days); community-onset CDI (CO-CDI) was a secondary outcome. Both were defined as LabID events using criteria from the CDC’s National Healthcare Safety Network (NHSN).^4^ Under NHSN rules, tests of asymptomatic patients are not considered LabID events; a patient who has a positive asymptomatic *C. difficile* test upon hospital admission remains eligible for a subsequent CO-CDI or HO-CDI event.

To assess unintended impacts of *C. difficile* screening, we analyzed unit-level enteral vancomycin days of therapy per 1,000 days present as a proxy for CDI treatment or prophylaxis and vancomycin-resistant *Enterococcus* (VRE) LabID events per 10,000 patient-days.^4^

For all analyses involving monthly rate data, we used interrupted time series models adjusted for repeated measures to evaluate differences in intercept and slope between the baseline and intervention periods. Analytic models were appropriate to count data (ie, Poisson or negative binomial) and included offset variables to account for monthly variation in patient census.

To assess patient-level outcomes, we created a retrospective cohort that included every asymptomatic *C. difficile* carrier (screen-positive) identified during the first year of intervention, plus non-carriers (screen-negative) selected in a 1:2 ratio based on hospital length of stay and admission date. Between carriers and non-carriers, we compared the following outcomes within the first fourteen days of hospitalization by calculating the relative risk using Fisher’s exact test: clinically significant diarrhea (ie, three or more unformed stools documented per day), symptomatic *C. difficile* testing, and receipt of enteral vancomycin therapy.

Statistical analyses were performed using SAS 9.4 software (SAS Institute, Cary, NC). This study was reviewed by the Rush University institutional review board and considered exempt, with informed consent waived.

## Results

During the study period, there were 4,205 patient admissions to the HO/HCT unit (2,121 during baseline and 2,084 during intervention). During the baseline period, there were 525 symptomatic *C. difficile* PCR tests performed, of which 85 (16.2%) were positive. During the intervention period, 519 symptomatic and 1,286 asymptomatic *C. difficile* PCR tests were performed, of which 55 (10.6%) and 66 (5.1%) were positive, respectively.

**Figure 1.**
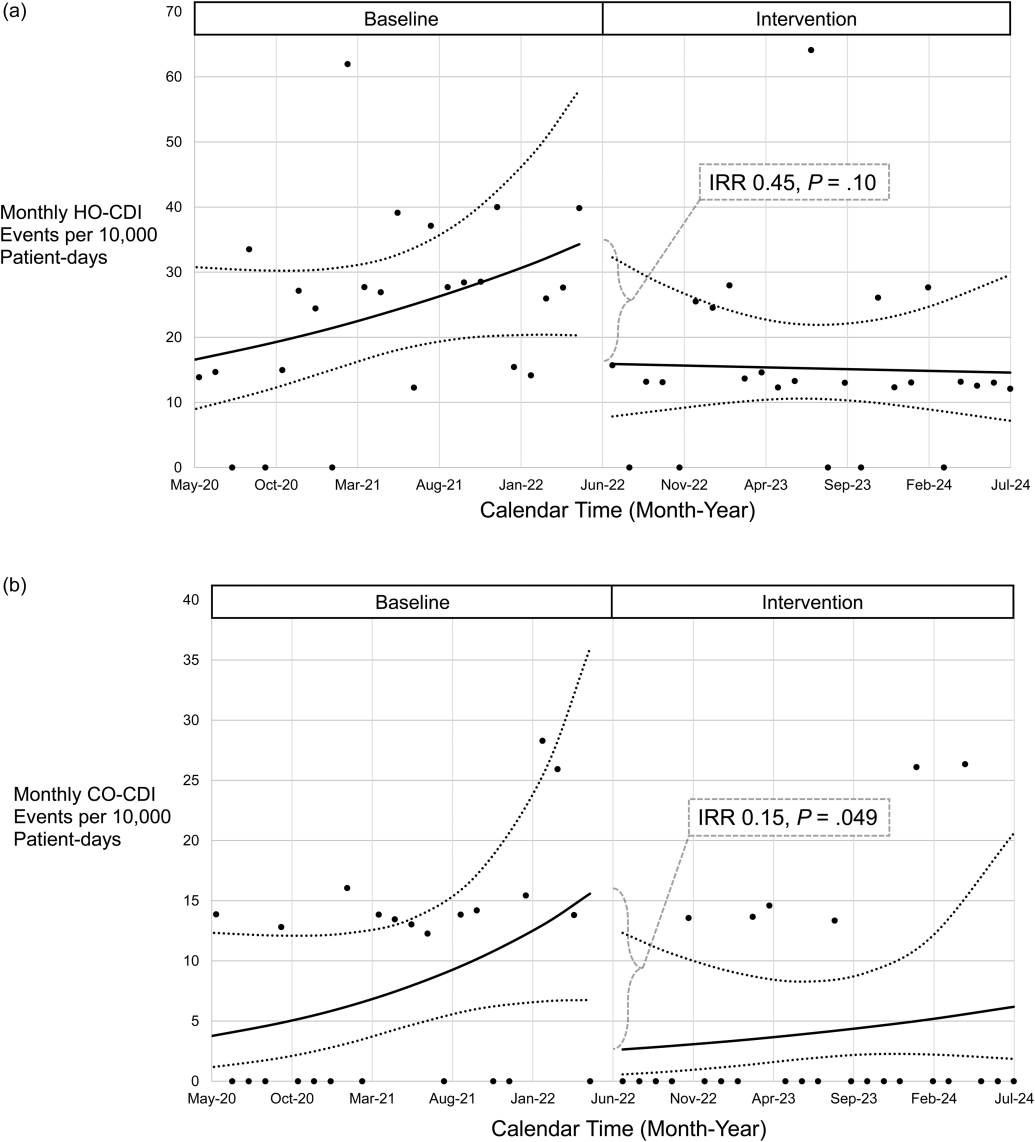
Figure 1 long description. Monthly hospital-onset [a] and community-onset [b] C*lostridioides difficile* infection (CDI) incidence per 10,000 patient-days. Baseline: symptomatic CDI testing only. Intervention: symptomatic CDI + admission *C. difficile* testing. Incidence rate ratio (IRR) refers to changes in intercept between baseline and intervention modeled rates. There were no significant changes in slope for either model presented.

The intervention period was associated with a non-significant decline in adjusted HO-CDI incidence compared to baseline period (incident rate ratio [IRR] 0.45, *P* = .10, Figure 1a). CO-CDI incidence was significantly lower in the intervention period, compared to baseline (IRR 0.15, *P* = .049, Figure 1b). The IRRs for enteral vancomycin days of therapy and VRE events were 1.01 (*P* = .97) and 3.70 (*P* = .11), respectively.

In a cohort of patients stratified by admission asymptomatic screening result, *C. difficile* carriers were equally likely to develop diarrhea as non-carriers; however, carriers were less likely to receive *C. difficile* symptomatic testing and more likely to receive enteral vancomycin (Table 1).

**Table 1.** Outcomes during hospitalization by *Clostridioides difficile* carriage status on admission, retrospective cohort

	Carriers (n = 25)	Non-carriers (n = 50)	Relative Risk	*P* value
Developed diarrhea^a^				
Hospital days 1–3, n (%)	2 (8)	3 (6)	1.33	.99
Hospital days 4–14, n (%)	5 (20)	10 (20)	1.00	.99
Underwent symptomatic testing, n (%)	0 (0)	10 (20)	0.00	.03
Received enteral vancomycin^b^, n (%)	6 (24)	2 (4)	6.00	.01

*Note:* ^a^Documentation of ≥3 unformed stools in 1 day.

^b^Enteral vancomycin could be administered for either the indication of prophylaxis or treatment.

Fidaxomicin was available as a restricted antibiotic for *C. difficile* infection treatment, requiring infectious diseases approval; no patient in this cohort analysis received fidaxomicin.

## Discussion

Admission screening for asymptomatic *C. difficile* colonization among patients admitted to the HO/HCT unit was associated with a non-significant decrease in HO-CDI rate and a significant decrease in CO-CDI rate. During the intervention period, screen-positive patients (carriers) developed diarrhea at comparable rates to screen-negative patients (non-carriers). However, carriers were less likely to be tested for symptomatic CDI during hospitalization and more likely to receive enteral vancomycin therapy (as prophylaxis or treatment) than their non-carrier counterparts.

Several prior studies have reported a decrease in HO-CDI rates associated with asymptomatic admission screening. Cho *et al*. found an 80% decrease in the HO-CDI rate on an HCT unit after implementation of stool-based *C. difficile* admission screening and isolation of carriers.^5^ Collison *et al*. reported a 62% decrease in LabID events with hospitalwide admission *C. difficile* screening of inpatients, including patients admitted to the oncology unit.^6^

Although *C. difficile* admission screening may reduce HO-CDI rates through isolation precautions and judicious use of CDI-associated medications in screen-positive patients,^7^ our findings indicate it may also do so by altering provider testing practices. If asymptomatic screening primarily reduced patient-to-patient *C. difficile* transmission, we would expect a greater decrease in HO-CDI rates (reflecting nosocomial transmission) than in CO-CDI rates (which reflect acquisition prior to admission). Instead, we found that admission screening had a greater impact on reducing CO-CDI incidence than on HO-CDI incidence. Furthermore, we found that *C. difficile* carriers were much less likely to undergo CDI testing than non-carriers during hospital days 1−3 and 4−14 (eligibility periods for CO-CDI and HO-CDI, respectively), even though rates of clinically significant diarrhea were similar in both groups. Collectively, these findings indicate that both CO-CDI and HO-CDI metrics—LabID events defined exclusively by a positive *C. difficile* test result—are susceptible to variation in provider testing practices influenced by admission screening outcomes.

Although we detected a trend toward higher VRE incidence during the intervention period, we did not detect any increase in unit-level enteral vancomycin use and only a minority of confirmed carriers received enteral vancomycin in our cohort analysis.

Our study has several limitations. First, patients who were admitted to a non-HO/HCT unit (eg, intensive care) and later transferred to the HO/HCT unit were ineligible for screening. Second, perirectal swabbing may be less sensitive than stool-based testing for *C. difficile* detection.^8^ For both limitations, false-negative categorization of *C. difficile* colonized patients at time of admission could bias the intervention effect towards the null. Lastly, our study may not be generalizable to facilities that incorporate routine enzyme immunoassay *C. difficile* toxin testing.

In summary, admission screening for asymptomatic *C. difficile* colonization in HO/HCT patients influenced both HO-CDI and CO-CDI rates, partly by shaping subsequent provider testing decisions. These findings highlight that laboratory-defined quality metrics can be impacted by admission screening through both intended and unintended effects.

## Supporting information

10.1017/ice.2026.10442.sm001 Lucky et al. supplementary material Lucky et al. supplementary material

## Supplementary material

The supplementary material for this article can be found at https://doi.org/10.1017/ice.2026.10442.

## Figure 1 Long description

Panel A: A line graph shows the monthly hospital-onset Clostridioides difficile infection (HO-CDI) events per 10,000 patient-days from May 2020 to July 2024. The x-axis represents calendar time in months and years, and the y-axis represents the number of events. The graph is divided into two periods: Baseline and Intervention. During the baseline period, the number of events increases over time. During the intervention period, the number of events decreases initially but then starts to increase again. An annotation indicates an incidence rate ratio (IRR) of 0.45 with a P-value of 0.10. Panel B: A line graph shows the monthly community-onset Clostridioides difficile infection (CO-CDI) events per 10,000 patient-days from May 2020 to July 2024. The x-axis represents calendar time in months and years, and the y-axis represents the number of events. The graph is divided into two periods: Baseline and Intervention. During the baseline period, the number of events increases over time. During the intervention period, the number of events decreases significantly. An annotation indicates an incidence rate ratio (IRR) of 0.15 with a P-value of 0.049.

Navigate back to Figure 1.

## References

[1] Larrainzar-Coghen T , Rodríguez-Pardo D , Barba P , et al. Prognosis of *Clostridium difficile* infection in adult oncohaematological patients: experience from a large prospective observational study. Eur J Clin Microbiol Infect Dis 2018;37:2075–2082. 30073433 10.1007/s10096-018-3341-4

[2] Sheth PM , Douchant K , Uyanwune Y , et al. Evidence of transmission of Clostridium difficile in asymptomatic patients following admission screening in a tertiary care hospital. PLoS One 2019;14:e0207138. 30742636 10.1371/journal.pone.0207138 PMC6370182

[3] Lin MY , Stein BD , Kothadia SM , et al. Impact of mandatory infectious disease specialist approval on hospital-onset *Clostridioides difficile* infection rates and testing appropriateness. Clin Infect Dis 2023;77:346–350. 37157903 10.1093/cid/ciad250

[4] Centers for Disease Control and Prevention. NHSN: Multidrug-resistant organism & Clostridioides difficile infection (MDRO/CDI) module. 2025. https://www.cdc.gov/nhsn/pdfs/pscmanual/12pscmdro_cdadcurrent.pdf. Accessed June 30, 2025.

[5] Cho J , Seville MT , Khanna S , Pardi DS , Sampathkumar P , Kashyap PC. Screening for *Clostridium difficile* colonization on admission to a hematopoietic stem cell transplant unit may reduce hospital-acquired *C difficile* infection. Am J Infect Control 2018;46:459–461. 29169936 10.1016/j.ajic.2017.10.009

[6] Collison M , Murillo C , Marrs R , et al. Universal screening for *Clostridioides difficile* at an urban academic medical center. Infect Control Hosp Epidemiol 2021;42:351–352. 32959739 10.1017/ice.2020.428

[7] Gilboa M , Meltzer E , Barda N , et al. Impact of asymptomatic *Clostridioides difficile* carriage screening on antibiotic stewardship among hospitalized patients. Infect Control Hosp Epidemiol 2025;46:944–947. 40740008 10.1017/ice.2025.10213

[8] Lee MM , Reske KA , Hink T , et al. Relationship between Clostridioides difficile stool concentration, nucleic acid amplification test results, and environmental contamination. Open Forum Infect Dis 2026;13:ofag121. 41929670 10.1093/ofid/ofag121 PMC13043071

