# Supplementary material for: Impact of admission screening on Clostridioides difficile infection rates in a hematology-oncology and hematopoietic cell transplant unit
Source: Infect Control Hosp Epidemiol. 2026 Apr 28;47(7):741–3. doi: 10.1017/ice.2026.10442 (PMC13222759; doi:10.1017/ice.2026.10442)
Supplement: Lucky et al. supplementary material [file S0899823X26104425sup001.docx]

**Supplemental Material**

Manuscript Title: “Impact of Admission Screening on *Clostridioides difficile* Infection Rates in a Hematology-Oncology and Hematopoietic Cell Transplant Unit”

Authors: Christine W. Lucky, MD, MPH^1a^, Lahari Thotapalli, MPH^1^, Laura K. Rusie, ScM^1^, Yoona Rhee, MD, MS^1^, Michael E. Schoeny, PhD^2^, Nicole A. Kraut, MSN, RN^3^, Alexandra Seguin, DNP, FNP-BC^3^, Brian D. Stein, MD^4^, Raul I. Rodriguez, MD^1b^, Mary K. Hayden, MD^1^, Michael Y. Lin, MD, MPH^1,3^

**Supplemental Figure 1**: Algorithm for *C. difficile* testing for patients admitted to the hematology-oncology hematopoietic cell transplant unit (14E), presented during kick-off education to providers


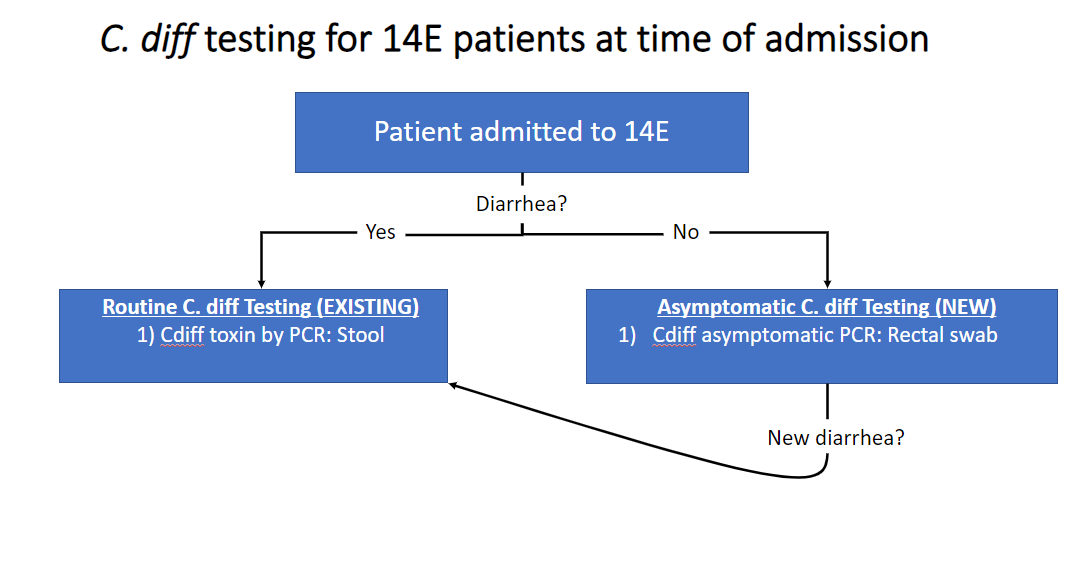


**Supplemental Figure 2 (A, B)**: *Clostridioides difficile* asymptomatic surveillance test results displayed to providers with embedded education.

**Panel A:** *C. difficile* asymptomatic screen, negative test result


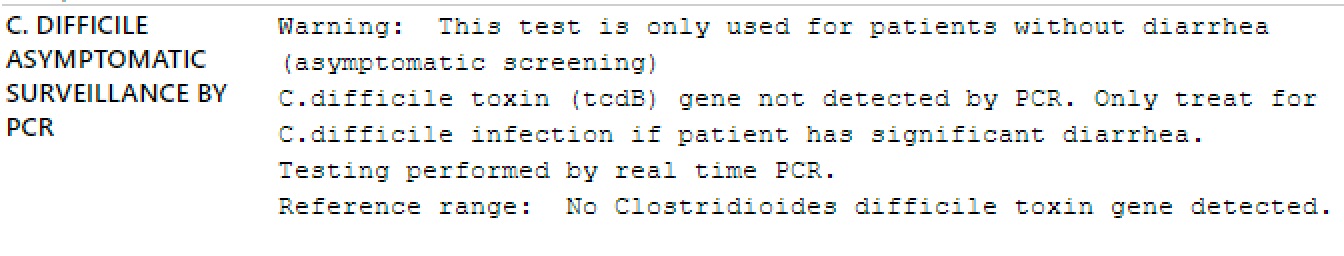


**Panel B:** *C. difficile* asymptomatic screen, positive test result


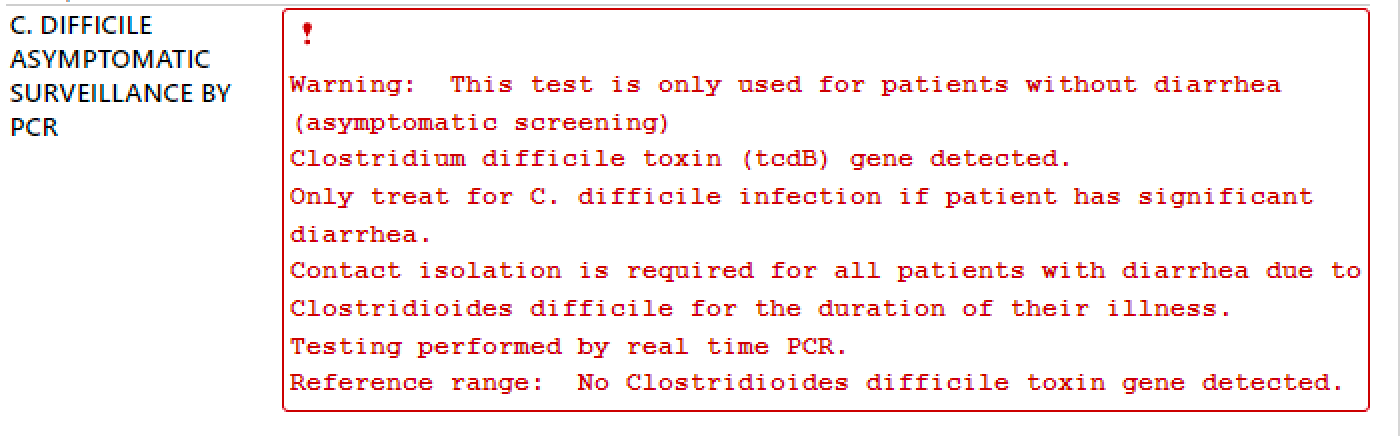


Note: Testing education was provided as shown to discourage unnecessary antibiotic treatment for *C. difficile* and to advise isolation precautions.
